# 抑癌基因*PTEN*与非小细胞肺癌相关性研究进展

**DOI:** 10.3779/j.issn.1009-3419.2014.03.13

**Published:** 2014-03-20

**Authors:** 亚楠 李, 洪武 王

**Affiliations:** 1 063009 唐山，河北联合大学 Hebei United University, Tangshan 063009, China; 2 100028 北京，北京煤炭总医院肿瘤内科 Department of Medical Oncology, China Meitan General Hospital, Beijing 100028, China

肺癌是当今世界严重威胁人类健康和生命的恶性肿瘤，也是世界范围内最常见的人类恶性肿瘤之一，发病率和死亡率呈逐年上升趋势，是发病率和死亡率最高的肿瘤之一。其中非小细胞肺癌（non-small cell lung cancer, NSCLC）约占肺癌总数的80%-85%^[1, 2]^，主要为鳞癌和腺癌两种亚型。虽然NSCLC在放疗、化疗、手术治疗方面取得了一定进展^[3, 4]^，但整体预后较差，5年生存率不足15%^[5]^。NSCLC发生、演变及预后过程是多个癌基因和抑癌基因共同参与的多步骤、多阶段、多基因改变并且有序的过程，在肺癌的发生、发展中起至关重要作用的有癌基因的激活和抑癌基因的失活、DNA修复基因的突变、生长因子信号转导通路和细胞周期调节的异常^[6]^。近年来，随着对肿瘤发病机制等各方面研究的不断深入以及分子生物学的飞速发展，目前人类已发现了多种原癌基因和抑癌基因，磷酸酶及张力蛋白同源基因（phosphatase and tensin homology deleted on chromosome ten, *PTEN*）是继p53后新发现的肿瘤抑癌基因，对PTEN研究不仅在于对其分子结构、功能、检测方法、抑癌机制等方面有重大意义，而且在对探索PTEN与NSCLC临床病理特征的关系方面也有非常重要作用。本文就其相关研究新进展综述如下。

## 1 *PTEN*基因的发现、鉴定及命名

早在1997年由美国Li等^[7]^三个研究小组等先后运用不同方法从人类第10q23.3染色体位点上分离、克隆、研究得到了同一种抑癌基因，从不同角度对其进行命名，分别命名第10号染色体缺失的PTEN、多种进展期癌中发生突变的基因1（mutated inmultiple advanced cancer 1, MMAC1）、TGF-β调节的上皮细胞富含的磷酸酶（TGF-β regulated and epitheliat cell-enriched phosphatase, TEP1），*PTEN*/*MMAC1*/*TEP1*基因均定位为第10q23.3染色体，且编码的蛋白质也为同一种蛋白质，表明3个基因为同一种基因，故*PTEN*亦可称*MMAC1*或*TEP1*，现该基因统称为*PTEN*。

## 2 PTEN的分子结构特点

*PTEN*基因定位于染色体10q23.3区，全长200 kb，由9个外显子和8个内含子组成，mRNA全长5.5 kb，其cDNA含有1, 209个核苷酸组成的开放阅读框架，编码着由403个氨基酸组成、分子量为47, 000的蛋白质。PTEN蛋白中与抑癌功能相关的结构由氨基端（N端）磷酸酯酶结构域、脂质结合的C2结构域和有50个氨基酸残基组成的羧基端（C端）结构域这3个区域共同组成^[8]^。在PTEN蛋白的氨基酸序列中，N端与细胞骨架蛋白张力蛋白（tensin）、神经触突泡转运相关辅助蛋白（auxilin）具有高度同源性，并含有一个蛋白酪氨酸磷酸酶（prote in tyrosine phosphatase, PTP）的催化结构域。其中辅助蛋白与神经突触小泡的运输密切相关，张力蛋白参与细胞的聚集与粘附，在肿瘤细胞的浸润、新生血管生成及转移中发挥一定作用。PTEN蛋白具有双特异性磷酸酶活性，磷酸酶催化结构域具有催化D3位脱磷酸的作用；Myers等^[9]^研究发现对丝氨酸、苏氨酸和酪氨酸的磷酸基起脱磷酸作用，是PTEN发挥肿瘤抑制活性的主要功能区域。脂质结合C2结构域与PTEN的磷酸酶结构域紧密相连，且C2区具有结合膜磷酯作用，能以Ca^2+^非依赖性方式使PTEN与细胞膜磷脂结合，参与PTEN催化结构域在细胞膜的正确有效定位和体内细胞的信号转导^[10]^。羧基端结构域包括两个PEST（350-375, 379-396）序列和尾去的一个PDZ结构域^[11]^，PTEN羧基端无磷酸酶，但PTEN蛋白羧基端尾部PEST序列酪氨酸、丝氨酸/苏氨酸残基磷酸化和PDZ结构域蛋白质之间的蛋白-蛋白的作用对调节其自身的稳定性和自身酶活性有重要作用，并且能改变PTEN对蛋白激酶B（protein kinase B, PKB）的调控，从而抑制细胞增殖、粘附、迁移^[12]^。

## 3 PTEN的抑癌作用机制和功能

PTEN作为第一个同时具有脂质和蛋白双重特异性磷酸酶活性的肿瘤抑癌基因，可参与细胞周期的调节和抑制肿瘤细胞的增殖、粘附、转移、血管生成及促进细胞的凋亡、分化、衰老等多种生理和病理活动。PTEN抑癌的作用机制主要包括3条途径：PI3K途径、FAK途径以及MAPK途径^[13]^。

### 3.1 PI3K途径

普遍认为PTEN抑制肿瘤发生的主要功能是依靠脂质磷酸酶活性，脂质磷酸酶能使磷酸肌醇通路的脂质脱磷酸，干扰磷脂酰肌醇-3-激酶/丝苏蛋白激酶（phosphatidylinositol 3-kinase/serine-threonine kinase, PI3K/Akt）/PKB信号转导^[14]^。PTEN作为脂质磷酸酶可以催化磷酯酰肌醇3, 4, 5-三磷酸（phosphatidylinositol 3, 4, 5-triphosphate, PIP3）去磷酸化为磷酯酰肌醇4, 5-二磷酸（phosphatidylinositol 3, 4, 5-triphosphate, PIP2），阻断PI3K-Akt/PKB信号转导途径，使细胞周期阻滞在G_1_期或促使细胞凋亡。PI3K是Akt/PKB信号转导上游的调节激酶，当刺激信号通过细胞表面生长因子受体酪氨酸激酶传递，使PI3K活化时，PI3K通过其3位羟基磷酸化，使PIP2生成PIP3，PIP3在体内积聚。PIP3作为某些生长因子如胰岛素、表皮生长因子等细胞内的第二信使，可吸引含有PH结构功能域的磷酸肌醇依赖性蛋白激酶Ⅰ和Akt/PKB使之激活，促进细胞生长，抑制其凋亡^[10, 15]^。

### 3.2 FAK途径

局灶粘附激酶（focal adhosion kinase, FAK）是整合素介导的信号转导途径中一个至关重要的分子，介导细胞与细胞外基质的粘附，整合素通过与细胞外基质作用而被活化，导致FAK酪氨酸磷酸化水平增高，并增加了其磷酸激酶的活性。*PTEN*基因N端序列与整合蛋白转导复合体中的细胞骨架蛋白有较高同源性，张力蛋白参与细胞的聚集与粘附，其突变失活常与部分肿瘤的恶性进展有关，提示PTEN可能参与调节细胞的粘连、新血管生成、迁移。PTEN可通过几种不同的途径对FAK进行调节，通过与FAK直接作用，使FAK/P130的酪氨酸水平降低。PTEN脂质磷酸酶活性具有调节FAK和Shc的磷酸化，使FAK去磷酸化而失活，抑制Shc磷酸化来调节细胞与细胞外基质的相互作用，从而影响细胞的迁移、粘连、侵蚀和血管形成能力的各个方面^[16]^。

### 3.3 MAPK途径

PTEN蛋白对丝裂原激活的蛋白激酶（mitogen-activated protein kinase, MAPK）途径上游中的细胞外信号转导激酶（extracellular signal-regulated kinase, ERK）、Ras的活化进行抑制还可抑制接头蛋白Shc的磷酸化水平和Ras的活动，亦还能对ERK/通过下调Ras介导的MAPK信号途径进行负调节，通过抑制介导MAPK有丝分裂信号向胞核传导，从而负调控细胞生长^[8]^。另外，PTEN蛋白的N端与张力蛋白高度同源，其过度表达可抑制肿瘤细胞的转移，PTEN可阻止细胞周期素D1（cell cycle- periodin D1, cyclin D1）聚集，使其下调，抑制细胞周期进展。*PTEN*基因还能通过抑制胰岛素受体底物-1（insulin recepter subtrate-1, IRS-1）的磷酸化从而抑制胰岛素生长因子-1（insulin-like growth facter-1, IGF-1）对MAPK的激活，使细胞生长周期阻滞于G_1_期，抑制PIP3去磷酸化，从而阻止细胞生长及促进细胞凋亡^[12, 16]^。MAPK途径在细胞转化和细胞周期调控中都起着重要的作用。

## 4 *PTEN*基因检测常用方法

### 4.1 *PTEN*基因突变检测常用技术

近年来随着分子生物学技术的发展，基因突变检测方法多种多样，这些方法各有优劣。现将常用的检测方法简述如下。

#### 4.1.1 直接测序法

直接测序法是最直接的、可检测已知和未知突变的而且也是目前被广泛应用于基因突变检测的一种方法^[17]^，王笑影^[18]^应用直接测序法和ARMS法同时检测168例NSCLC患者*EGFR*基因的突变，检测结果显示突变患者61例，直接测序法检测出32例，敏感性为52.5%，ARMS法检测出57例，敏感性为93.4%（*P*＜0.001）。直接测序法灵敏度相对较低，且较易出现交叉污染。

#### 4.1.2 基于即时PCR基础上的方法（如ARMS法）^[19]^

该法实现扩增时闭管操作，操作简单，无需产物的后处理，极大程度地避免了扩增产物的污染且检测灵敏度明显高于直接测序法^[18]^，可检测样品中含量低至1.0%的突变基因，但只能检测已知突变。

#### 4.1.3 聚合酶链反应-单链构象多态性分析（PCR-SSCP）法

该法简单快速，因而被广泛用于未知基因突变的检测^[20]^，同时该法也是检测抑癌基因*PTEN*突变最常应用的方法，但认为该法只能做为一种检测突变的方法，Guo等^[21]^要测定突变的准确突变位点和类型，还需进一步测序，且该法还可能会存在1%的假阳性率^[22, 23]^。

### 4.2 PTEN蛋白表达的检测方法

免疫组织化学（immunohistochemistry, IHC）中链霉菌性生物素蛋白-过氧化物酶连接（streptavidin-perosidase, SP）法是最常用的检测PTEN蛋白表达的方法，同时因其价格低廉、特异性高、操作简便、工作量较小而被广泛应用于临床和科研工作中，IHC（SP法）的最大优势是能检测到肿瘤特异性抗原，而且不会破坏细胞和组织的结构特征，但该方法敏感性低。据大量文献报道，SP法广泛用于检测多种肿瘤的PTEN蛋白的表达情况，舒红等^[24]^用SP法对肺癌组织中PTEN蛋白的表达情况进行检测、吕汕群等^[25]^用免疫组化SP检测宫颈癌组织中PTEN蛋白的表达。

随着肿瘤发病率的不断增长，现在越来越多的标本进行*PTEN*基因的检测，但到目前为止仍无敏感特异的方法。寻找有效可行的方法准确检测*PTEN*基因是未来的发展方向。

## 5 PTEN在NSCLC中的表达及其临床病理意义

### 5.1 PTEN蛋白在NSCLC组织中的表达结果

*PTEN*基因失活可表现为突变、缺失及PTEN mRNA或蛋白的低表达、失表达、甚至不表达，人类多种肿瘤中存在PTEN的失活，PTEN的失活导致其抑癌作用丧失，提示*PTEN*基因的失活在肿瘤的发生、发展及预后中具有重要的作用。脑胶质母细胞瘤、卵巢癌、前列腺癌、甲状腺癌、肺癌等不同类型肿瘤*PTEN*的突变率和突变方式、多形性及PTEN蛋白失表达的机制可不同，可表现为杂合性缺失（loss of heterozygosity, LOH）、纯合子缺失、表达缺失、甲基化、基因沉默等，而目前多数研究表明，PTEN发生异常的主要形式为LOH或纯合子缺失。在不同肿瘤中*PTEN*基因缺失发生的早晚也不相同，大多数发生在肿瘤的后期。Shu等^[24]^研究报道在肺癌中，PTEN蛋白表达完全失活多发生在肿瘤的晚期，此时抑癌作用消失，肿瘤更具有浸润性和远处转移能力，提示PTEN蛋白表达缺失对肺癌的进展具有一定的促进作用。张蕾等^[26]^应用聚合酶链反应及杂合性丢失分析法检测PTEN的杂合性丢失，结果显示肺癌中存在PTEN杂合性丢失。Kohon等^[27]^应用免疫组化方法研究40株肺癌细胞系，15株小细胞癌中6株*PTEN*基因纯合性缺失，2株有错义突变和无意义突变，25株NSCLC中2株纯合性缺失，也显示小细胞癌中的*PTEN*基因失表达高于NSCLC。Soria等^[28]^研究报道125例早期NSCLC患者中有30例（24%）PTEN阴性，其中20例PTEN阴性的标本有7例（35%）发生甲基化。尽管NSCLC中发生*PTEN*基因突变是少见的，但PTEN蛋白表达缺失或下降是相对较为普遍的^[27]^。大量研究结果认为PTEN蛋白表达发生异常主要为LOH，但Whang等^[29]^研究了10例晚期前列腺癌，仅1例纯合性丢失，其余9例中有5例显示不表达或表达降低，经用甲基化试剂处理，使无表达细胞恢复了表达，故认为造成PTEN蛋白表达缺失部分是源于该基因的甲基化。关于PTEN表达异常的主要方式及其他表达方式或成分在肺癌发生、发展中是否存在异常，还需进一步研究。

### 5.2 PTEN蛋白的表达与NSCLC临床病理特征的关系

由于国内外有关PTEN与NSCLC的研究不多，结果也不完全一致。到目前为止，大量研究发现，PTEN蛋白低水平表达与NSCLC的分化程度、临床病理分期、淋巴结转移、生存率及预后密切相关，而与患者的年龄、性别、肿瘤大小、部位、组织学类型、吸烟指数、身体状况及体重减轻无关。

#### 5.2.1 PTEN表达与NSCLC分化程度及病理分期、分级的关系

PTEN蛋白表达随NSCLC组织分化程度的降低、临床分期的升高而降低，表明PTEN蛋白表达水平低的肺癌恶性程度相对增高。张红蕊等^[30]^观察到，PTEN蛋白表达与非小细胞肺癌TNM分期相关，在Ⅰ期-Ⅱ期比Ⅲ期-Ⅳ期阳性表达率明显增高，差异有统计学意义。Goncharuk等^[31]^也观察到PTEN的表达与肿瘤分期、分化程度明显相关。在高中分化组、Ⅰ期-Ⅱ期组的阳性表达率比低分化组、Ⅲ期-Ⅳ期阳性表达率明显增高，预示着在肿瘤进展过程中伴随着PTEN的下调，PTEN的下调与生存期的缩短明显相关，可作为判断患者病情预后不良的重要指标，说明PTEN蛋白表达缺失或下降在NSCLC发生、发展中起着重要的作用。

#### 5.2.2 PTEN表达与NSCLC淋巴结转移的关系

PTEN表达下调与肺癌的浸润、转移密切相关，PTEN蛋白低表达或表达阴性的肺癌患者更容易发生淋巴结转移，与张建海等^[32]^研究的结论基本一致。其原因可能是因为PTEN可使FAK去磷酸化，失去对FAK介导的细胞浸润、转移和生长的抑制作用^[24]^，从而使得癌组织更容易发生淋巴结转移。

#### 5.2.3 PTEN表达与NSCLC患者预后的关系

众多研究表明，PTEN的蛋白表达缺失对NSCLC的发展有促进作用是NSCLC发生发展过程中的晚期事件之一。随肺癌恶性程度的增高及远处转移的出现PTEN阳性表达率明显降低。Bepler等^[33]^观察到，PTEN表达率高的NSCLC患者比表达率低的患者生存期长、疾病复发转移较晚。张建海等^[33]^报道PTEN蛋白表达阳性患者平均生存时间较阴性患者明显延长，其5年生存率明显高于PTEN蛋白表达阴性患者，说明PTEN蛋白表达阳性率高的肺癌患者往往预后较好。因此大量资料证实NSCLC分化程度、临床分期、有无淋巴结转移等均是影响NSCLC患者病情严重程度及预后的重要因素，而PTEN蛋白表达高低却是判断NSCLC病情严重程度及预后的一个独立预测因子^[34]^。

## 6 小结及展望

*PTEN*被称为继p53时代后突变率最高的“肿瘤抑制基因”，作为到目前为止被发现的第一个具有双重特异性磷酸酶活性的抑癌基因，PTEN通过诱导细胞分化、凋亡、阻滞细胞周期进程、调节细胞增殖、抑制肿瘤血管生长及细胞的粘附等多种途径发挥其抑制肺癌发生发展的进程，成为肺癌研究的新焦点。*PTEN*基因失活与NSCLC发生发展、组织分化、临床病理分期、淋巴结转移及生存期等生物学过程有着极其重要关联，并可成为判断NSCLC患者病情严重程度及预后不良的指标之一。因此深入研究PTEN与NSCLC的内在联系将对NSCLC的早期诊断、有效治疗和预后判断起重要的指导作用。同时，随着人们对PTEN研究的逐步加深，PTEN的抑癌机制、PTEN与其他癌基因、抑癌基因之间的相互作用，及其与细胞凋亡和凋亡相关基因之间的相互关系会进一步明朗，这将为临床监测、预后评估、药物疗效、开发新的抗肿瘤药物及指导临床治疗提供新思路，也为肿瘤细胞耐化疗药的研究、肺癌PTEN基因的靶向药物治疗提供新途径。
